# From blame to learning: implementing a just culture program for head nurses and its impact on silent behavior and error reporting among staff nurses

**DOI:** 10.1186/s12912-025-04265-5

**Published:** 2026-01-24

**Authors:** Fawzia Mohamed Mohamed Badran, Marwa Abd El Rahman Gaber Khalifa, Howaida Moussa Elghannam, Eman Hassan Mohamed Ali

**Affiliations:** 1 Nursing Administration Department, Faculty of Nursing, Ain Shams University, Cairo, Egypt; 2 Nursing Administration, Faculty of Nursing – Modern University for Technology and Information (MTI), Cairo, Egypt

**Keywords:** Just culture, Head nurses, Silent behavior, Error reporting, Patient safety, Staff nurses

## Abstract

**Background:**

Establishing a Just Culture in healthcare is essential to improving patient safety, encouraging open communication, and reducing fear-based silence among staff. Nurse leaders play a critical role in modeling and promoting Just Culture principles. However, the impact of targeted Just Culture programs for head nurses on staff behavior remains underexplored.

**Aim:**

This study aimed to assess the effect of implementing a Just Culture program for head nurses on silent behavior and error reporting among staff nurses at Ain Shams Specialized Hospital, Egypt.

**Methods:**

A quasi-experimental, one-group pretest–posttest design was conducted at Ain Shams Specialized Hospital. The study sample included 70 head nurses and 400 staff nurses. Data were collected using five tools: (1) a demographic and work-related data sheet; (2) Head Nurses’ Just Culture Knowledge Assessment; (3) Just Culture Assessment Tool (JCAT); (4) the Silent Behavior Scale for Nurses (SBSN); and (5) the Staff Nurses’ Error Reporting Questionnaire. Statistical analysis was conducted using SPSS version 20. Paired sample t-tests were used to compare pre- and post-program scores. The Chi-square test was applied to assess changes in categorical variables (e.g. knowledge levels). A p-value ≤ 0.05 was considered statistically significant. Cohen’s d was used to determine effect sizes.

**Results:**

Following the educational intervention, head nurses demonstrated statistically significant improvements in their knowledge and perceptions of Just Culture across all measured domains (p < 0.001). The overall Just Culture knowledge score increased markedly from a mean of 61.0 ± 9.5 pre-program to 88.5 ± 6.2 post-program, with large effect sizes (Cohen’s d 1.3) indicating a substantial impact. Similarly, perception showed a significant improvement following the intervention, increasing from a pre-program mean of 72.7 ± 10.2 to a post-program mean of 111.2 ± 8.6 (t = 13.95, p < 0.001). Among staff nurses, there was a significant reduction in silence behaviors, as evidenced by decreased scores on the Silent Behavior Scale for Nurses (SBSN) (p < 0.001), alongside notable increases in frequency and accuracy of error reporting post-intervention (p < 0.001). Effect sizes for all staff nurse outcomes were large (Cohen’s d 1.5), reflecting meaningful improvements in communication and error reporting culture.

**Conclusion:**

Implementing a Just Culture educational program for head nurses led to significant improvements in managerial understanding and support for safety culture. These changes positively influenced staff nurses’ communication behaviors and error reporting practices. Findings suggest that leadership-based Just Culture programs can be an effective strategy for enhancing patient safety outcomes in hospital settings.

**Clinical trial registration:**

Not applicable.

## Introduction

In today’s complex healthcare systems, ensuring patient safety remains a critical priority. Central to this aim is the development of a culture that promotes open communication, particularly regarding medical errors and adverse events [1]. However, many healthcare organizations still operate within a blame culture, where individuals are often punished for mistakes. This punitive approach discourages healthcare providers, especially nurses, from reporting incidents which undermine efforts to improve safety and organizational learning. The fear of punishment, loss of professional reputation, and emotional distress can lead nurses to remain silent, even when patient harm is at stake [2].

Blame culture is widely recognized as a significant obstacle to patient safety, primarily due to the fear it instills in healthcare staff around reporting errors and admitting fault [3]. For nurses who serve as frontline caregivers and are often the first to witness or be involved in medical errors this culture can be particularly detrimental [4]. Frontline health professionals frequently hesitate to report adverse events due to distress of penalty or blame, compounded by a lack of confidence that such reporting will result in meaningful improvements. This fear may stem from potential negative consequences, including malpractice lawsuits, loss of patient trust, emotional responses from patients or families, and even the risk of job termination [5].

A persistent culture of blame often leads to the attribution of errors to individual failings rather than recognizing them as symptoms of broader systemic issues [6]. This blame-centric approach discourages healthcare professionals from reporting mistakes due to fear of punishment, thereby hindering opportunities for organizational learning and improvement. Such a culture not only impedes the identification and rectification of underlying system flaws but also adversely affects the well-being of healthcare providers, who may experience increased stress and burnout [7].

This blame culture environment reinforces a cycle of fear, silence, and preventable harm. The recognition of these adverse outcomes has led to a shift in thinking within healthcare leadership, giving rise to more balanced and constructive approaches such as just culture. In response to the ongoing challenge of a blame-oriented culture in healthcare, leaders are increasingly turning to the just culture framework. This model shifts the focus from assigning blame to fostering learning, promoting accountability in a fair and consistent manner [8]. A key factor in the successful implementation of this model is leadership particularly head nurses who serve not only as policy enforcers but also as moral exemplars within clinical units [1].

Transitioning to a “just culture” is essential for enhancing patient safety and fostering a more supportive work environment [7]. Ultimately, building a culture grounded in voluntary error reporting and continuous learning is essential for achieving sustainable safety improvements [5]. By adopting a just culture, healthcare organizations can eliminate the fear of repercussions, empowering frontline staff to view errors as opportunities for improvement rather than grounds for punishment [9].

Just culture is a concept first introduced in the aviation industry in the 1980s. As originally defined by James Reason, it is “a collective understanding between blameless and blameworthy actions” [5, 10]. Just culture refers to an environment in which members of an organization are encouraged to exchange critical safety-related information feedback and support, ultimately increasing trust among members [11]. Also, just culture’ is described as the processes intended to achieve a just decision regarding the action to be taken in respect of individuals involved in either adverse safety incidents or near misses. [12].

An essential part of just culture is creating a culture of psychological safety to allow staff to have the confidence to share safety concerns and report adverse events without fear of being subject to retribution. [1]. This not only helps in identifying potential risks but also promotes a culture of trust and collaboration within the team. For head nurses, who are often at the forefront of implementing these cultural changes, establishing trust and fairness within their teams is crucial. They must model behaviors that promote openness, such as acknowledging mistakes and focusing on solutions, rather than accusing individuals [13].

Enacting this culture in a hospital environment enhances the incident reporting mechanism by creating a culture where staff are going to feel comfortable to report patient safety incidents. Enhanced organizational control also helps to strengthen staff capacity in managing issues with patient safety incidents [14, 15]. While engaging in such exchanges, a just culture allows organizations to identify if an individual’s behavior is indicative of human error, at-risk behavior, or reckless behavior [16]. Studies indicate that when nurse leaders engage in transparent communication and active listening, staff members are more likely to report adverse events, leading to better patient outcomes and higher overall morale [17].

In many settings, nurses are reluctant to speak up in the face of adverse events, especially if they involve physicians or higher-level staff [18]. When nurses fear retribution or punishment, they are less likely to speak up or report these incidents [19]. This silence can create a “second victim” phenomenon, where the emotional toll of involvement in a mistake is exacerbated by a lack of organizational support. Furthermore, punitive responses can foster a toxic work environment, characterized by distrust, defensive communication, and low moral [17]. Nurses may perceive that their voices are undervalued or dismissed, leading them to remain silent rather than challenge the status quo or escalate concerns. These power dynamics create barriers to effective communication and hinder the reporting of safety issues [18].

Unreported errors or near misses deprive the organization of critical opportunities to learn from those incidents and implement corrective actions. This failure to address systemic defects can lead to repeated errors, ultimately putting patients at greater risk of harm [20]. Moreover, silent behavior contributes to the undervaluation of nursing expertise within healthcare teams. When nurses are discouraged from speaking up, it not only harms patient safety but also diminishes the role of nursing staff in contributing to decision-making processes. This can lead to low morale, job dissatisfaction, and burnout, as nurses may feel undervalued or powerless in their roles [21, 22].

When errors are concealed, healthcare organizations miss critical opportunities to analyze system errors and implement preventive strategies. As a result, the same types of errors may be repeated, compromising the safety and quality of care [23]. Understanding why medical errors happen is important to learn from them and prevent them from occurring in the future. However, there is a long history of blaming and shaming after a medical error has occurred, making healthcare workers less likely to report errors due to potential repercussions. [24].

Adverse events defined as any unintended injury or complication caused by management in healthcare - represent critical information about system failures that, if managed, can prevent injury in the future. Yet, there is still a general reluctance to report adverse events, often motivated by concerns of being disciplined, embarrassment to the organization, or possible legal action. To help facilitate reporting to address such concerns and promote a strong reporting culture in their organization, healthcare organizations should plan to transition to a just culture by developing standardized procedures for evaluating adverse events and developing reporting systems. [1].

Transitioning to a “just culture” is essential for enhancing patient safety and fostering a more supportive work environment [7]. The implementation of a just culture significantly reduces the fear of retaliation, one of the most significant factors that contribute to silent behavior in healthcare settings. When nurses feel supported by their leadership and know that their concerns will be addressed in a constructive manner, they are more likely to report adverse events [25]. A supportive environment enhances psychological safety, which is crucial for the development of trust and transparency in healthcare teams. As a result, nurses are more willing to come forward with safety concerns, leading to a greater volume of error reports and a more accurate picture of safety risks within the organization [4].

For the effective implementation of just culture, it is essential that head nurses provide comprehensive education and training on the principles of just culture to their staff. This training should not only focus on the differentiation between human errors, at-risk behavior, and reckless behavior but should also emphasize the role of nursing staff in fostering a safety culture [26]. Head nurses can organize workshops, seminars, and simulations to engage their team in practical learning exercises that mirror real-world clinical situations [27].

By ensuring that staff are educated on the expectations of just culture, head nurses can reduce confusion or misinterpretation of the framework. It is also important for head nurses to reinforce these concepts, as consistent education ensures that just culture remains an integral part of the organizational culture. Periodic refresher courses and feedback sessions can help reinforce the understanding of just culture over time, ensuring it becomes embedded in everyday practice [28–30].

Adopting just culture has profound implications for nursing practice. Reducing silent behavior and increasing error reporting creates a safer environment where nurses can focus on providing quality care without the burden of fear or guilt associated with mistakes. This cultural shift allows for better teamwork, more effective communication, and a stronger commitment to patient safety across the nursing team [31]. Additionally, the ability to learn from errors contributes to the professional development of nurses, as they are encouraged to reflect on their practice and improve their clinical skills [32].

The long-term adoption of just culture has also been linked to improved organizational performance. Healthcare organizations that invest in cultivating a just culture experience greater employee engagement and job satisfaction, as staff feel valued and respected within the organization. Nurses report higher levels of psychological safety and a stronger sense of professional fulfillment when they are encouraged to report errors without fear of payback [4].

Furthermore, as a just culture encourages continuous learning, healthcare organizations become more adaptable and responsive to changes in healthcare delivery [6]. Organizations that prioritize learning from errors are better positioned to innovate and improve their practices over time. As nurses and other healthcare professionals develop their skills through this ongoing learning process, they become more competent and confident in their roles, leading to better care quality and greater patient satisfaction [5–34].

### Significance of the study

In healthcare settings, particularly in high-stakes environments such as hospitals, the culture surrounding error reporting is critical to ensuring patient safety and fostering organizational learning. However, many institutions continue to operate within blame-oriented cultures that discourage transparency and promote silence among staff nurses. This silence, often driven by fear of punishment, emotional distress, or perceived futility, undermines the quality of care and obstructs the identification of systemic issues that require intervention [3]. The just culture model presents a constructive alternative by shifting the focus from individual punishment to organizational learning from errors. This study is significant in that it examines the role of head nurses in leading this cultural transformation within healthcare settings. Through supporting fairness, trust, and psychological safety, head nurses can create space for staff nurses to report adverse events and have open, positive, and productive conversations without fear of reprisal [6, 8].

Supporting this perspective, an integrated review by Barkell and Snyder [35] emphasized the importance of implementing organizational interventions that strengthen culture, increase the reporting of errors and near-misses, and foster continuous learning. The World Health Organization [36] has outlined strategies to clearly distinguish between human error and negligence as a foundation for promoting a just culture. It defines just culture as an approach that acknowledges the complexity of healthcare environments and attributes most patient safety failures to system-level weaknesses rather than individual fault.

However, despite these recommendations, there is a noticeable gap in the literature regarding the implementation and effectiveness of comprehensive just culture interventions across various healthcare settings in Egypt. Specifically, there is a lack of empirical studies that assess how hospital managers at different levels adopt and integrate just culture principles into their daily practices and how these practices influence nurses’ willingness to report medication errors. Moreover, El-Gazar [37] emphasizes the importance of improving all dimensions of Just Culture, there is limited research exploring the multifaceted interventions that address these dimensions and their impact on error reporting behaviors among nurses.

Therefore, your research could address these gaps by investigating how hospital managers in various Egyptian healthcare settings implement just culture principles and evaluating the effectiveness of comprehensive interventions that target all dimensions of just culture in promoting nurses’ intention to report medication errors. Given this, implementing a just culture program specifically tailored for head nurses is essential in shifting healthcare environments from blame-based systems to learning-oriented cultures. Evidence indicates that when nurse leaders adopt and model just culture principles, they help create a psychologically safe environment, where nurses feel comfortable reporting errors without concern for punitive action.

These insights reinforce the necessity for structured leadership development programs. Implementing a just culture program for head nurses has the potential to strengthen transparency, accountability, and psychological safety across nursing teams. As such, this study will evaluate the impact of a just culture training program for head nurses at Ain Shams University Hospital. The primary aim is to reduce silent behavior and enhance error reporting among staff nurses, addressing a critical gap in leadership-based patient safety strategies. Ultimately, the findings will contribute evidence-based recommendations to improve nursing practice, support a culture of safety, and advance patient outcomes.

### Aim of the study

This study aimed to evaluate the effect of implementing a just culture program for head nurses on staff nurses’ silent behavior and error reporting practices.

### Research hypotheses

Based on the study objectives, the following hypotheses were formulated:

1. Educational intervention will significantly enhance head nurses’ knowledge and perceptions of just culture; post-intervention compared to pre-intervention.
2. The intervention will significantly reduce silent behavior among staff nurses, reflected post-intervention.
3. The frequency and accuracy of error reporting among staff nurses will significantly increase following the educational intervention.
4. There will be significant correlations between improvements in head nurses’ Just Culture knowledge and perceptions and changes in staff nurses’ silent behavior and error reporting practices.

## Methods

### Design

A quasi-experimental one-group research design with pre-post assessment was employed to conduct the study.

### Setting

The study was conducted at Ain Shams Specialized Hospital, a prominent healthcare institution affiliated with Ain Shams University in Cairo, Egypt, which is affiliated with the Ministry of Health and Population (MOHP), with a bed capacity (*n* = 800) beds. The hospital serves a diverse and expansive population, offering specialized care across a variety of medical disciplines. The hospital consists of four buildings, each with three floors, and it provides a wide range of medical services across various specialties. Hospital specialties included Oncology, Neurosciences (Neurology & Neurosurgery), Organ Transplantation, Nephrology & Urology, Gastroenterology & Hepatology, Pediatrics & Neonatology, Obstetrics & Gynecology, Emergency & Trauma Care, Intensive Care Units (ICUs), Radiology & Imaging, Pathology, Geriatrics, Rehabilitation Medicine specialty.

As a key tertiary referral center, it plays a crucial role in providing advanced healthcare services to patients from across Egypt and neighboring countries. This hospital took some serious steps to advance the requirements of the General Authority for Health Accreditation and Regulation (GAHAR) about patient safety. This research has the ability to positively influence both the workforce and patient care quality at the hospital level, contributing to the benefit of individuals in the local or regional communities the institution serves.

### Sampling

The participants in the study included all head nurses, as well as the staff nurses working in the study settings during the study time. Their numbers were 400 staff nurses and 70 head nurses. The study included two different samples or groups: head nurses and staff nurses. These samples included nurses who were working in the previously mentioned settings at the time of the study, regardless of their age, gender, qualifications, or years of experience. The sample size for head nurses was 70, and all of them were included in the study. For staff nurses, the sample size was calculated to detect an improvement in the scores of knowledge, just perception and change silent behavior with a moderate effect size (0.43) according to Brydges [38]. Using the G*Power software package, Version 3.1.9.4 [39], at a 95% level of confidence and 80% power, the sample size was 400 nurses after accounting for a 10% dropout rate.

### Data collection tools

Data were collected using five tools: Demographic and work-related data sheet; Head Nurses’ Just Culture Knowledge Assessment; Just Culture Assessment Tool (JCAT); Silent Behavior Scale for Nurses (SBSN); and Staff Nurses’ Error Reporting Questionnaire.

### Tool I: Demographic and work-related data sheet

It was developed by the researchers, and it included (age, gender, educational levels, years of nursing experience, years of experience in leadership roles, attend any workshops about just culture).

### Tool II: Head nurses’ just culture knowledge assessment

It was developed by researchers based on relevant literature Van Baarle et al. [16], Fencl et al. [40], and its validity and reliability were established prior to use. It was administered both before and after the intervention. The questionnaire consisted of 20 items, categorized into four key dimensions: (1) understanding of non-blame culture, (2) error reporting, (3) improvement of patient safety, and (4) leadership. The purpose of the questionnaire was to evaluate head nurses’ knowledge and understanding of these essential components of just culture, which is critical for fostering a supportive and transparent environment in healthcare settings.

Scoring system:Subjects’ responses to the knowledge questionnaire were scored dichotomously, with each correct answer assigned a score of 1 and each incorrect answer assigned a score of 0. The questionnaire consists of 20 items, resulting in a total possible score ranging from 0 to 20. Knowledge levels will be categorized based on percentage thresholds as follows: scores below 14 ( < 70%) will be considered unsatisfactory, scores between 14 and 16 (70%–80%) will be considered satisfactory, and scores of 17 or higher (80%) will be classified as highly satisfactory. These cutoff points were adapted from Ayesh et al. [41] to provide a meaningful interpretation of the participants’ knowledge levels. The reliability of the data collection tools was assessed as its internal consistency by using Guttman split-half Coefficient test. Head Nurses’ Just Culture Knowledge Assessment was 0.910.

### Tool III. Just Culture Assessment Tool JCAT

It was used to assess head nurses’ perceptions of just culture within their work environment. The tool was originally developed by Petschonek et al. [42] and later adapted for use in healthcare settings by El-Gazar et al. [37] and its validity and reliability were established prior to use. It was administered both before and after the intervention. It was comprised 27 items distributed across six dimensions: feedback and communication (3 items; e.g., Management does a good job of sharing information about events), openness of communication (5 items; e.g., Supervisors respect suggestions from staff members), balance (5 items; e.g., When an event occurs, the follow-up team looks at each step in the process to determine how the event happened), quality of the event reporting process (5 items; e.g., “I’m given time to enter event reports during work hours), continuous improvement (4 items; e.g., “The hospital sees events as opportunities for improvement”), and trust (5 items; e.g., “I trust that the hospital will handle events fairly).

**Scoring system:**Responses were measured using a 7-point Likert-type scale ranging from 1 (strongly disagree) to 7 (strongly agree). Total scores for the 27-item Just Culture Assessment Tool (JCAT) ranged from 27 to 189, with higher scores reflecting more positive perceptions of Just Culture within the hospital. This scoring method has been previously utilized and validated in healthcare settings [37]. The reliability of the data collection tools was assessed as its internal consistency by using Guttman split-half Coefficient test. Just Culture Assessment Tool (JCAT) was 0.903.

### Tool VI: Silent behavior scale for nurses (SBSN)

It included two parts:

Part I: This was for nurse’s demographic data included age, gender, educational levels, years of nursing experience, years of experience, attend any workshops about patient safety.

Part II: It was developed by researchers based on Edmondson [43] and Zou et al. [44], it was a 9-item tool designed to assess staff nurses’ silent behavior in healthcare settings. It is organized into three dimensions: Fear-Based Silence, Ineffective Communication, and Professional Silence.

• Fear-Based Silence: This dimension evaluates how fear of negative consequences prevents nurses from speaking up. An example item is: I hesitate to speak up when I notice a safety issue because I fear negative consequences.

• Ineffective Communication: This dimension explored barriers in communication systems that hinder the reporting and addressing of concerns. An example item is: When a safety issue arises, I don’t know the proper channels to report it.

• Professional Silence: This dimension assessed the unspoken expectation to remain silent in certain situations. An example is I often remain silent about issues or errors because it’s not seen as my role to speak up.

**Scoring system:** Nurse responses measured on a 5-point Likert scale: 1 (Never), 2 (Rarely), 3 (Sometimes), 4 (Often), and 5 (Always). The total score for the SBSN ranged from 9 to 45, with higher scores indicating more frequent silent behavior. The scores are categorized as follows: a low level (9–17) indicates minimal silent behavior, suggesting that the nurse feels comfortable speaking up; a moderate level (18–32) reflects occasional barriers to communication, where some fear or uncertainty may influence silence; and a high level (33–45) indicates significant silent behavior, reflecting strong barriers such as fear of consequences or ineffective communication systems that may hinder the reporting of safety concerns. The total score is further interpreted based on percentage thresholds: a high level is 75%, a moderate level is between 60–75%, and a low level is < 60%, following the categorization established by Zou et al. [44]. The reliability of the data collection tools was assessed as its internal consistency by using Guttman split-half Coefficient test. Silent Behavior Scale for Nurses (SBSN) was 0.89.

### Tool VII. Error reporting questionnaire

It was developed by researchers based on Anderson & Webster [33], Aljabari et al. [24] and Singer et al. [45]. It was used to assess staff nurses’ regarding various aspects of error reporting. This questionnaire is composed of (27 items) subdivided into five dimensions including frequency, attitudes, barriers, feedback, and organizational support.

**Scoring system:** The responses of staff nurses on the Error Reporting Questionnaire were measured using a combination of Likert-type scales, tailored to the nature of each dimension. Most items were rated on a five-point Likert scale: 1 = Strongly Disagree, 2 = Disagree, 3 = Uncertain, 4 = Agree, and 5 = Strongly Agree. Items within the frequency of reported errors dimension was assessed using a three-point Likert scale: 1 = Never, 2 = Occasionally, and 3 = Always. For items related to the prevalence of nursing errors, a binary scoring system was applied, with Yes = 1 and No = 0.

The total score was calculated by summing responses across all 27 items. Based on percentage thresholds of the maximum possible score, error reporting levels was categorized as follows: low ( < 50%), moderate (50–70%), and high (70%). This classification approach follows the statistical guidance provided by Singer et al. [45] allowing for a structured interpretation of staff nurses’ engagement in error reporting practices. The reliability of the data collection tool was assessed its internal consistency by using Guttman split-half Coefficient test. Staff Nurses’ Error Reporting Questionnaire was 0.911.

### Validity

The content and face validity of the instruments were assessed by a jury panel comprising seven experts, including five professors and two assistant professors from the Nursing Administration Department, Faculty of Nursing, Ain Shams University, Cairo, Egypt. The instruments were evaluated for their applicability, comprehensiveness, and relevance to the study objectives. The experts were invited to review the proposed instruments and provide their feedback. Based on their recommendations, necessary modifications were made, including the addition and exclusion of specific items to enhance the clarity and appropriateness of the tools. To quantify content validity, the Content Validity Index (CVI) was calculated at both the item level (I-CVI) and the scale level (S-CVI). Items with an I-CVI of ≥0.78 were considered acceptable, while the S-CVI for the overall instruments exceeded 0.90, indicating excellent content validity. These results support the content robustness and appropriateness of the instruments for use in the current study context.

### Ethical considerations

We confirm our study was conducted in accordance with the principles outlined in the Declaration of Helsinki. The Scientific Research Ethical Committee of the Faculty of Nursing, Modern Technology Information University, gave ethical approval (Code number, FAN/174/2025). The Faculty of Nursing provided official letters to the designated hospital for permission to collect data. After informing each participant (head nurses and staff nurses) about the objectives and procedures of the study, informed written consent was obtained from them. All participants were assured of their right to refuse or withdraw from the study at any time. The confidentiality and anonymity of any information obtained was guaranteed through secured coded data. No actual or possible harm or pressure was anticipated from the investigation’s maneuvers.

### Pilot study

The pilot study was conducted on forty nurses, and seven head nurses who made up 10% of the main study sample. It was served to test the clarity of the tools and the applicability of the study. It was also served to estimate the time to conduct the interview. Based on the results of the pilot study necessary modifications would be made. The pilot sample was not included in the main sample to avoid contamination since the study type is intervention.

### Field work

The actual fieldwork of the study extended over a period of five months, and involved several key phases, including preparation, planning, implementation of the just culture training program and post program evaluation.

### Phase I (preliminary)

After securing official approvals for conducting the study, the researchers met with the director of hospital and head nurses of units to determine the most suitable time to collect data. The researchers were available five days a week, during morning and afternoon shifts to collect the data. The researchers met head nurses and nurses, explaining the purpose and nature of the study, and obtained written consent to participate in the study. Then, they were given the data collection tools, instructing them how to fill out. The researchers were present to answer any questions while the participants were filling out the forms The filled-out forms were handed back to the researcher to check their completeness. Collected data was considered baseline or pretesting.

### Phase II (program planning)

During this planning phase, the content of the program was developed based on a review of the current and past literature, using textbooks, scientific articles in magazines, and internet searches, in addition to the results of the pretest assessment (Head Nurses Knowledge Questionnaire, Just Culture Assessment Tool, Silent Behavior Scale, and Staff nurse’ error reporting questionnaire). Various instructional strategies were selected based on participants’ needs to meet the objectives and content of the training program. It was focused on providing participants with as much experience as possible. A suitable space/venue was reserved for the training program. The program schedule was prepared accordingly. It covered theoretical aspects and practical aspects regarding the topic of the study.

### Phase III (program implementation)

The just culture educational program consisted of eight structured sessions. The total duration of the intervention took (one month) at a frequency of two sessions per week, with each session lasting between 90 to 120 minutes. The sessions are designed to enhance head nurses’ competencies in fostering a culture of safety and accountability. Session topics was included: (1) orientation and introduction to Just Culture, (2) understanding the no-blame culture, (3) consequences of silent behavior, (4) foundations of error reporting, (5) strategies for improving patient safety, (6) leadership in Just Culture, (7) enhancing team communication and trust, and (8) practical application and reflection as illustrated in Table 1.

**Table 1:** Just culture educational program for head nurses – theory and practice overview

Session	Topic	Theory Content	Practical Activities
**1**	Orientation & Introduction to Just Culture	- Definition, principles, and evolution of just culture - Importance of leadership in culture change- Overview of program goals and structure	- Icebreaker: “What does safety culture mean to you?”- Reflection on past reporting experiences- Program orientation walkthrough
**2**	Understanding the No-Blame Culture	- Differences between human error, at-risk, and reckless behavior- Systems thinking approach- Psychological safety in reporting	- Case study analysis (error categorization)- Role-play: Giving non-blaming feedback- Group discussion on fear and barriers
**3**	Consequences of Silent Behavior	- Impact of silence in healthcare- Normalization of deviance- Ethical considerations in reporting failures	- Group activity: Ripple effects of silence- Simulation: Identifying silent behaviors- Reflective journaling on silence in practice
**4**	Foundations of Error Reporting	- Reporting systems and policies- Legal protections for reporters- Common barriers to reporting in nursing	- Practice: Fill out mock incident reports- Peer review: Report evaluation- Video analysis: Real-life incident case
**5**	Strategies to Improve Patient Safety	- High-Reliability Organization (HRO) principles- Models, Root cause analysis (RCA)- Proactive vs reactive safety approaches	- Conduct RCA in a team- Brainstorm safety improvements- Safety audit walkthrough in unit
**6**	Leadership in Just Culture	- Transformational leadership- Nurse leader’s role in culture shift- Coaching and mentoring for change	- Case study: Leadership response to error- Simulation: Accountability conversations- Leadership self-assessment and action plan
**7**	Enhancing Team Communication & Trust	- Communication tools: SBAR, closed-loop- Trust and psychological safety- Conflict resolution in teams	- Role-play: SBAR handoff scenarios- Team trust-building exercise- Simulation: Handling team miscommunication
**8**	Practical Application & Reflection	- Integration of just culture into daily practice- Sustaining change through leadership- Monitoring and evaluation of culture	- Group project presentations- Incident response simulation- Personal reflection & commitment plan

Each session incorporated both theoretical and practical components, utilizing lectures, case studies, simulations, and group discussions to reinforce learning. At the beginning of the first session, participants received a comprehensive orientation to the program’s objectives and structure. Feedback was solicited at the start of each session to assess comprehension and refine subsequent content delivery, ensuring responsiveness to participants’ learning needs.

The education strategies utilized during the program included mini-lectures, small group discussions, and lessons with practical application using role play, case scenarios, group reflection, and demonstration-re-demonstration. Educational media included PowerPoint presentations, posters, and flipcharts. Handouts were prepared by the researchers and distributed to the participants. The head nurses applied Just Culture principles in daily practice (handover meetings, incident reporting discussions, huddles, team briefings, and performance feedback), which was reflected in the study results (error reporting was increased and silent behavior was decreased).

### Phase IV (post program evaluation)

The effect of the program was evaluated through a posttest immediately after the end of the program implementation and after three months. This was done using the same data collection tools as in the pretest.

### Statistical design

Data entry and statistical analysis were done using the SPSS ‎‎20.0 statistical ‎software package. Quality control was done at the stages ‎of coding and data entry. Data ‎were presented using descriptive statistics ‎in the form of frequencies and percentages for ‎qualitative variables and ‎means and standard deviations for ‎quantitative variables. Cronbach’s alpha coefficient was ‎calculated to ‎assess the reliability of the developed tools through their ‎internal consistency. ‎Paired-samples t-tests were used to compare pre- and post-program scores for head nurses and staff nurses. Cohen’s d was calculated to estimate effect sizes, with thresholds of small (d < 0.5), medium (0.5 ≤ d < 0.8), and large (d ≥ 0.8). Statistical significance was considered at *p* < 0.05.

## Results

Table 2 presents the socio-demographic and professional characteristics of the studied head nurses (*n* = 70). The majority of participants were aged between 35 and 44 years (40.0%), with a mean age of 41.2 ± 7.8 years, indicating a mid-career professional cohort. Most were female (85.7%), which aligns with the gender distribution typically seen in the nursing workforce. Regarding educational qualifications, 60.0% of the head nurses held a bachelor’s degree, while 35.7% had a master’s degree, and a small proportion (4.3%) held a doctorate. The mean years of nursing experience was 17.6 ± 6.5 years, with 40.0% having ≥20 years of experience. In terms of leadership tenure, the mean duration was 7.9 ± 3.4 years, with 44.3% of participants serving in leadership roles for 5–10 years. Importantly, none of the participants had received formal training in just culture, highlighting a significant gap in professional development related to patient safety and organizational culture.

**Table 2:** Personal data for studied head nurses (*n* = 70)

Characteristic	Category	N	%	Mean ± SD
Age (years)	25–34	14	20.0%	41.2 ± 7.8
	35–44	28	40.0%	
	45–54	21	30.0%	
	≥55	7	10.0%	
Gender	Female	60	85.7%	
	Male	10	14.3%	
Education Level	Bachelor’s degree	42	60.0%	
	Master’s degree	25	35.7%	
	Doctorate	3	4.3%	
Years of Nursing Experience	< 10 years	11	15.7%	17.6 ± 6.5
	10–19 years	31	44.3%	
	≥20 years	28	40.0%	
Years in Leadership Role	< 5 years	25	35.7%	7.9 ± 3.4
	5–10 years	31	44.3%	
	10 years	14	20.0%	
Attended Just Culture Training	Yes	0	0.0%	
	No	70	100.0%	

Table 3 summarizes demographic and professional characteristics of the 400 staff nurses included in the study. The majority were in the 30–39 year age group (40.0%), followed by those aged 40–49 years (27.5%), with a mean age of 36.4 ± 8.6 years. Female nurses dominated the sample (80.0%), with males making up 20.0%. In terms of education, most nurses held a Bachelor’s degree (72.5%), while smaller proportions had a Master’s degree (15.0%) or Doctorate (2.5%); 10.0% held a Diploma. The distribution of nursing experience showed that nearly half (47.5%) had 5–14 years of experience, 30.0% had 15 or more years, and 22.5% had less than 5 years; the mean experience was 11.8 ± 5.9 years. With respect to years in the current role, 42.5% had served between 3 and 7 years, 37.5% less than 3 years, and 20.0% for more than 7 years, with a mean tenure of 5.4 ± 2.7 years. Notably, none of the staff nurses (0.0%) reported having attended Just Culture training.

**Table 3:** Personal data for studied staff nurses (*n* = 400)

Characteristic	Category	N	%	Mean ± SD
Age (years)	20–29	80	20.0%	36.4 ± 8.6
	30–39	160	40.0%	
	40–49	110	27.5%	
	≥50	50	12.5%	
Gender	Female	320	80.0%	
	Male	80	20.0%	
Education Level	Diploma	40	10.0%	
	Bachelor’s degree	290	72.5%	
	Master’s degree	60	15.0%	
	Doctorate	10	2.5%	
Years of Nursing Experience	< 5 years	90	22.5%	11.8 ± 5.9
	5–14 years	190	47.5%	
	≥15 years	120	30.0%	
Years in Current Role	< 3 years	150	37.5%	5.4 ± 2.7
	3–7 years	170	42.5%	
	7 years	80	20.0%	
Attended Just Culture Training	Yes	0	00.0%	
	No	400	100%	

Cohen’s d: Small effect size: d < 0.5, Medium effect size: 0.5 ≤ d < 0.8, Large effect size: d ≥ 0.8

The data in Table 4 demonstrate a statistically significant improvement in just culture knowledge among head nurses following the intervention across all assessed domains. Each dimension—including understanding of non-blame culture, error reporting, patient safety improvement, and leadership—showed substantial increases in mean scores with highly significant p-values ( < 0.001). Cohen’s d values ranged from 1.37 to 1.46, indicating very large effect sizes. Effect sizes were calculated using Cohen’s d to quantify the magnitude of differences between pre- and post-program scores and were interpreted according to Cohen’s guidelines, where d = 0.2 is considered small, 0.5 medium, and 0.8 large (Cohen [46]). These findings underscore the practical significance of the improvements observed. The overall knowledge score increased markedly from 61.0 to 88.5, with a large effect size (d = 1.68), confirming the program’s strong impact on enhancing nurse leaders’ understanding and application of Just Culture principles.

**Table 4:** Just culture knowledge assessment (pre- and post-program) for head nurses (*n* = 70)

Dimension	No. of Items	Pre-Program Mean ± SD	Post-Program Mean ± SD	t-value	P-value	Cohen’s d
1. Understanding of Non-Blame Culture	5	3.12 ± 0.88	4.40 ± 0.60	9.85	< 0.001 **	1.39
2. Error Reporting	5	2.98 ± 0.75	4.35 ± 0.58	10.32	< 0.001 **	1.46
3. Improvement of Patient Safety	5	3.25 ± 0.82	4.50 ± 0.55	9.70	< 0.001 **	1.37
4. Leadership	5	3.05 ± 0.90	4.42 ± 0.63	9.95	< 0.001 **	1.41
**Total Knowledge Score (out of 100)**	20	61.0 ± 9.5	88.5 ± 6.2	11.85	< 0.001 **	1.68

Cohen’s d: Small effect size: d < 0.5, Medium effect size: 0.5 ≤ d < 0.8, Large effect size: d ≥ 0.8

Table 5 presents a categorical analysis of knowledge levels before and after the intervention. The proportion of participants in the “Highly Satisfactory” category increased markedly from 5.7% at pre-test to 52.9% at post-test. Conversely, those classified as Unsatisfactory” decreased from 52.9% to 2.9%. These shifts reflect a substantial improvement in knowledge outcomes, supported by very large effect sizes (Cohen’s *d* = 1.90 and 1.79), indicating a practically meaningful and statistically significant impact of the educational program.

**Table 5:** Knowledge level changes and effect size at pre-test, post-Test(*n* = 70)

Knowledge Level	Pre-Program, n (%)	Post-Program, n (%)	Cohen’s d
Unsatisfactory ( < 70%)	37 (52.9%)	2 (2.9%)	1.90
Satisfactory (70–80%)	9 (12.9%)	11 (15.7%)	0.09
Highly Satisfactory (80%)	4 (5.7%)	37 (52.9%)	1.79

Cohen’s d:Small effect size: d < 0.5, Medium effect size: 0.5 ≤ d < 0.8, Large effect size: d ≥ 0.8

Table 6 Following the intervention, all just culture perception dimensions showed statistically significant improvements (*p* < 0.001). The large Cohen’s d effect sizes indicate that these changes are not only statistically significant but also practically meaningful, reflecting a substantial enhancement in head nurses’ perceptions across key areas including feedback and communication, openness, balance, event reporting quality, continuous improvement, and trust. These results demonstrate the program’s effectiveness in positively influencing leadership attitudes towards a Just Culture.

**Table 6:** Just culture perception scores before and after the program for head nurses (*n* = 70)

JCAT Dimension	No. of Items	Pre-Program Mean ± SD	Post-Program Mean ± SD	t-value	P-value	Cohen’s d
Feedback and Communication	3	8.6 ± 2.1	12.3 ± 1.6	9.04	< 0.001 **	1.28
Openness of Communication	5	13.8 ± 3.4	20.1 ± 2.5	10.15	< 0.001 **	1.43
Balance	5	14.2 ± 3.1	21.4 ± 2.1	11.22	< 0.001 **	1.59
Quality of Event Reporting Process	5	12.6 ± 3.2	19.7 ± 2.6	9.88	< 0.001 **	1.40
Continuous Improvement	4	10.1 ± 2.7	16.6 ± 1.9	10.05	< 0.001 **	1.42
Trust	5	13.4 ± 3.0	21.1 ± 2.2	11.70	< 0.001 **	1.65
**Total JCAT Score**	27	72.7 ± 10.2	111.2 ± 8.6	13.95	< 0.001 **	1.97

Cohen’s d: Small effect size: d < 0.5, Medium effect size: 0.5 ≤ d < 0.8, Large effect size: d ≥ 0.8

Table 7 illustrates the significant reduction in Silent Behavior Scale for Nurses (SBSN) scores among 400 nurses following the intervention. All dimensions—fear-based silence, ineffective communication, and professional silence showed statistically significant decreases (*p* < 0.001), indicating a marked decline in silence behaviors that can hinder effective communication and patient safety. The total SBSN score decreased from 31.8 to 19.7, reflecting a substantial overall reduction in silence-related behaviors. The effect sizes, as indicated by Cohen’s d values ranging from 1.70 to 2.89, demonstrate very large effects, underscoring the strong impact of the program in promoting open communication and reducing barriers to speaking up among nursing staff.

**Table 7:** Silent behavior Scale for nurses scores before and after the program (*n* = 400)

SBSN Dimension	No. of Items	Pre-Program Mean ± SD	Post-Program Mean ± SD	t-value (n = 400)	P-value	Cohen’s d
Fear-Based Silence	3	10.9 ± 2.6	6.4 ± 1.6	25.46	< 0.001 **	2.08
Ineffective Communication	3	10.3 ± 2.5	6.6 ± 1.7	23.91	< 0.001 **	1.73
Professional Silence	3	10.6 ± 2.7	6.7 ± 1.8	24.95	< 0.001 **	1.70
**Total SBSN Score**	9	31.8 ± 4.9	19.7 ± 3.3	26.98	< 0.001 **	2.89

Cohen’s d: Small effect size: d < 0.5, Medium effect size: 0.5 ≤ d < 0.8, Large effect size: d ≥ 0.8

Table 8 presents the pre- and post-program scores of the Staff Nurse Error Reporting Questionnaire (*n* = 400). The results reveal statistically significant improvements across all dimensions following the intervention (*p* < 0.001). Specifically, the frequency of reported errors increased substantially, reflecting more consistent reporting behavior. Positive shifts in attitudes towards error reporting and organizational support indicate enhanced nurse engagement and perceived backing for error reporting activities. Meanwhile, significant reductions in perceived barriers suggest fewer obstacles to reporting errors. Improvements in feedback processes also demonstrate a more responsive and supportive environment for post-intervention. Cohen’s d values ranged from 1.32 to 1.90, indicating very large effect sizes across all dimensions and the total score. These findings highlight the strong impact of the program on fostering a culture that encourages error reporting among staff nurses, which is critical for patient safety and quality improvement initiatives.

**Table 8:** Staff nurse error reporting scores before and after the program (*n* = 400)

Dimension	No. of Items	Pre-Program Mean ± SD	Post-Program Mean ± SD	t-value	P-value	Cohen’s d
Frequency of Reported Errors	6	1.45 ± 0.52	2.30 ± 0.41	28.10	< 0.001 **	1.68
Attitudes	7	3.12 ± 0.68	4.25 ± 0.50	32.45	< 0.001 **	1.80
Barriers	5	2.85 ± 0.72	1.95 ± 0.60	26.22	< 0.001 **	1.32
Feedback	5	2.90 ± 0.70	4.00 ± 0.55	30.10	< 0.001 **	1.60
Organizational Support	4	3.05 ± 0.75	4.10 ± 0.62	28.75	< 0.001 **	1.50
**Total Score**	27	13.37 ± 3.25	17.60 ± 2.90	35.80	< 0.001 **	1.90

## Discussion

This study aimed to evaluate the impact of a structured educational intervention for head nurses on their knowledge and perceptions of just culture and to examine its influence on staff nurses’ silent behavior and error reporting practices. The findings confirm that the intervention significantly improved head nurses’ understanding and attitudes toward just culture, reduced silent behaviors among staff nurses, and enhanced both the frequency and accuracy of error reporting following the program.

A statistically significant improvement in just culture knowledge was observed among head nurses’ post-intervention, with marked gains across all domains, including non-blame culture, error reporting, patient safety, and leadership. The overall knowledge score increased from 61.0 to 88.5, accompanied by a large effect size (Cohen’s *d* = 1.68) [46], indicating a meaningful and impactful change. These improvements are likely attributable to the program’s dual focus: content specifically tailored for nursing leaders and comprehensive coverage of core just culture principles. These improvements can be rationalized by the structure and content of the intervention, which was intentionally designed to address the specific learning needs of nurse leaders. By focusing on both theoretical foundations and practical application of just culture principles such as differentiating between types of errors, promoting fair accountability, and fostering a non-punitive environment. The program equipped participants with both the knowledge and confidence required to implement cultural change within their teams.

This aligns with findings from Ayesh et al. (2023) [41], who reported similar improvements in nurse leaders’ knowledge and application of non-blame culture following intervention. Collectively, these findings emphasize the effectiveness of structured educational programs in cultivating a just culture within healthcare settings, which may lead to improved error reporting, greater communication openness, and enhanced patient safety outcomes. However, existing literature suggests that sustaining such improvements requires ongoing training and reinforcement to firmly embed cultural change into routine practice (Hanifi et al. 2018) [47]. Overall, this body of evidence supports the critical role of educational initiatives in advancing healthcare workers’ understanding and application of just culture principles. The categorical shift in knowledge levels particularly the increase in participants achieving “Highly Satisfactory” scores further underscores the program’s effectiveness. These findings respond to gaps identified by Murray et al. (2023) [48], who emphasized the need for knowledgeable leadership as a precursor to cultural transformation.

A statistically significant improvement was observed in head nurses’ perceptions of just culture following the educational intervention, with the most notable gains in the dimensions of trust (Cohen’s *d* = 1.65) and balance (Cohen’s *d* = 1.59). These improvements are likely due to the program’s emphasis on leadership behaviors that foster psychological safety, fair accountability, and open communication skills that are essential for building trust and enabling leaders to appropriately differentiate between types of errors. By enhancing leaders’ ability to respond to mistakes in a fair and non-punitive manner, the intervention likely contributed to shifting long-standing cultural norms. This interpretation is supported by Yusof and Razali (2024) [3], who emphasized that leadership commitment to fairness and transparency is essential for promoting trust within healthcare teams, and by Logroño et al. (2023) [5], who highlighted variability in nurses’ perceptions of trust and balance as key indicators of organizational culture. Within the Egyptian context, where punitive responses and lack of feedback have historically hindered open communication, these shifts represent a crucial step toward creating safer, more communicative clinical environments (Mostafa et al. 2022) [49]. These enhancements also reflect worldwide evidence emphasizing the importance of leadership in shaping a just culture. Pozzobon et al. (2024) [50] stated that healthcare leaders are key to “establishing and maintaining a just culture to support the reporting of and learning from patient safety incidents.” This emphasizes that leadership-directed interventions are crucial to building a just culture of fairness, transparency, and psychological safety across a variety of healthcare systems.

The intervention yielded a statistically significant and substantial reduction in silent behaviors among staff nurses, as measured by the Silent Behavior Scale for Nurses (SBSN), with marked decreases observed across all its dimensions—fear-based silence, ineffective communication, and professional silence reflected in the total SBSN score declining sharply from 31.8 to 19.7 and accompanied by very large effect sizes (Cohen’s *d* = 2.08, 1.73, and 1.70, respectively), The significant reduction in silent behaviors among staff nurses can also be explained by the intervention’s role in strengthening organizational culture at the unit level. By empowering head nurses with effective communication skills and fostering a leadership style that prioritizes empathy, fairness, and support, the program cultivated an environment where nurses felt psychologically secure to express concerns without fear of negative consequences.

This enhanced climate of trust and mutual respect has likely encouraged nurses to overcome traditional barriers such as hierarchical pressures and fear of reprisal, which often contribute to silence in clinical settings. Furthermore, the intervention’s focus on clarifying expectations around accountability and promoting non-punitive error management helped normalize open dialogue and reinforced staff confidence that raising issues would lead to constructive outcomes rather than punishment. This interpretation aligns closely with Mostafa et al. (2022) [49], who identified fear and punitive organizational cultures as primary inhibitors to transparent communication, and is further reinforced by Van Baarle et al. (2022) [16], who emphasized that leadership-driven initiatives to foster open communication and accountability serve as critical mechanisms to promote cultural transformation within healthcare settings, ultimately facilitating a climate where nurses feel empowered to raise concerns, contribute to patient safety, and engage in proactive error reporting without fear of negative consequences.

The program also positively influenced error reporting practices among staff nurses. Post-intervention scores revealed statistically significant improvements in all dimensions of error reporting (*p* < 0.001), including a marked increase in reporting frequency. These findings suggest the development of a psychologically safer environment, where staff felt more empowered to report incidents without fear. This aligns with Chegini et al. (2020) [51], who found that nurses’ intention to report errors was strongly associated with leadership support and a non-punitive organizational culture. Additionally, consistent with Massah et al. (2021) [52], our results affirm that educational interventions targeting leadership can drive staff engagement and proactive safety behaviors. When leadership fosters supportive, fair responses to error, it not only encourages reporting but also enables effective root cause analysis and organizational learning (Murray et al. 2023; Yusof & Razali, 2024) [3, 48].

One of the enduring challenges in implementing Just Culture is balancing non-punitive learning with accountability for unsafe or reckless behavior. This study’s intervention successfully addressed that balance by equipping nurse leaders with conceptual tools to apply fair, situational judgment in response to clinical incidents. This approach reflects the balanced Just Culture framework described by Murray et al. (2023) [48], ensuring both psychological safety and organizational responsibility. In doing so, the intervention supports a broader cultural shift one that transitions from a punitive, pathological safety culture” (Moshiri et al. 2025) [11] to a productive and resilient one where mistakes are seen as opportunities for learning rather than punishment.

Finally, the findings highlight the broader significance of embedding Just Culture principles into leadership education. When head nurses are equipped with knowledge and skills to model fairness, encourage open communication, and respond constructively to errors, the impact extends beyond individual attitudes it reshapes organizational norms. As Yusof and Razali (2024) [3], argue, such cultural transformation is essential for supporting both patients and healthcare workers, including ‘second victims’ of clinical errors. This study reinforces the importance of sustained, leadership-focused interventions to drive systemic improvements in safety culture across healthcare systems.

Taken together, the results of this study demonstrate that leadership-focused educational interventions can serve as a powerful catalyst for cultivating a just culture within healthcare settings. By improving head nurses’ knowledge and perceptions, and by positively influencing staff behavior and reporting practices, the intervention addressed key barriers to communication and safety often found in clinical environments. These changes not only reflect individual growth among nursing leaders but also signal broader cultural transformation at the unit level. To sustain these gains, ongoing leadership development, institutional commitment, and policy level integration of Just Culture principles are essential. Such efforts will be critical in fostering psychologically safe, transparent, and high-performing healthcare environments.

## Conclusion of the study

The educational just culture program produced significant improvements in head nurses’ knowledge, perceptions related to just culture principles. These enhancements were associated with meaningful reductions in silence behaviors and increased error reporting among staff nurses, indicating a positive shift toward a more transparent and psychologically safe work environment. The study confirms that targeted, leader-focused education is an effective strategy for advancing just culture within healthcare settings, addressing critical gaps in knowledge and attitudes that hinder patient safety efforts. By fostering balanced accountability and non-punitive responses to errors, nurse leaders can act as catalysts for cultural change that supports both staff well-being and improved clinical outcomes. Future initiatives should build upon these findings by expanding educational programs and integrating systemic support to sustain and broaden the impact on healthcare safety culture.

### Implications of the research

This research highlights the vital role of targeted educational intervention in improving head nurses’ knowledge and perceptions of just culture, which in turn positively influences staff behavior and patient safety practices. Healthcare organizations should prioritize tailored Just Culture training for leadership positions, as knowledgeable leaders are essential for fostering a non-punitive environment that encourages open communication and accurate error reporting.

The findings suggest that educational interventions can effectively address barriers such as fear of blame and stigma, which commonly inhibit error reporting. By empowering nurse leaders with a balanced understanding of accountability, healthcare institutions can cultivate a culture of trust and psychological safety, essential components for sustainable patient safety improvements.

Furthermore, the behavioral changes demonstrated among staff nurses underscore the importance of integrating leadership development with organizational support systems. Policymakers and healthcare administrators should consider incorporating just culture principles into ongoing professional development and institutional policies to facilitate long-term cultural transformation and safer clinical environments. Also, further research is suggested across multiple hospitals or healthcare settings to see if the impact of a Just Culture program on silent behavior and error reporting is consistent in different organizational contexts.

### Limitation of the study

First, one of the limitations is that it was relying on self-report tools. Second, single-site data collection hinders the generalizability of the findings.

## Data Availability

Due to confidentiality concerns, the data and materials used in the current research cannot be made publicly available. However, they are available from the corresponding author upon reasonable request.
